# Investigating the effects of mobile bottom fishing on benthic carbon processing and storage: a systematic review protocol

**DOI:** 10.1186/s13750-024-00348-z

**Published:** 2024-10-15

**Authors:** Stacey L. Felgate, John Aldridge, Stefan G. Bolam, Sarah Breimann, Emil de Borger, Jolien Claes, Jochen Depestele, Graham Epstein, Clement Garcia, Natalie Hicks, Michel Kaiser, Jack H. Laverick, Gennadi Lessin, Finbarr G. O’Neill, Sarah Paradis, Ruth Parker, Ryan Pereira, Alex J. Poulton, Claire Powell, Craig Smeaton, Paul Snelgrove, Justin Tiano, Johan van der Molen, Sebastiaan van de Velde, Marija Sciberras

**Affiliations:** 1 The Lyell Centre for Earth and Marine Science, Heriot-Watt University, Edinburgh, EH14 4AP UK; 2 Centre for Environment, Fisheries, and Aquaculture Science (CEFAS), Pakefield Road, Lowestoft, Suffolk, NR33 0HT UK; 3 Plymouth Marine Laboratory, Prospect Place, Plymouth, PL1 3DH UK; 4 Department of Estuarine and Delta Systems, Royal Netherlands Institute of Sea Research (NIOZ), Landsdiep 4, 1797 SZ ‘t Horntje, Texel, The Netherlands; 5 Flanders Research Institute for Agriculture, Fisheries, and Food (ILVO), Jacobsenstraat 1, Oostende, 8400 Belgium; 6 Department of Biological Sciences, University of Victoria, Victoria, British Colombia V8P 5C2 Canada; 7 School of Life Sciences, University of Essex, Colchester, CO4 3SQ UK; 8 Department of Mathematics and Statistics, University of Strathclyde, Glasgow, Lanarkshire, G1 1XH UK; 9 National Institute of Aquatic Resources (DTU AQUA), Technical University of Denmark, North Sea Science Park, Hirtshals, 9850 Denmark; 10 Department of Earth Sciences, Geological Institute, ETH Zürich, Sonneggstrasse 5, Zürich, 8092 Switzerland; 11 School of Geography and Sustainable Development, University of St Andrews, Irvine Building, St Andrews, KY16 8LG UK; 12 Department of Biology, Memorial University of Newfoundland, 232 Elizabeth Ave, St. John’s, NL, A1B 3X9 Canada; 13 Wageningen Marine Research, Wageningen University and Research, IJmuiden, The Netherlands; 14 Department of Geosciences, Environment & Society, Université Libre de Bruxelles, Brussels, Belgium; 15 Operational Directorate Natural Environment, Royal Belgian Institute of Natural Sciences, Brussels, Belgium

**Keywords:** Benthic carbon, Marine sediments, Demersal trawling, Resuspension, Anthropogenic disturbance

## Abstract

**Background:**

Marine sediments represent one of the planet’s largest carbon stores. Bottom trawl fisheries constitute the most widespread physical disturbance to seabed habitats, which exert a large influence over the oceanic carbon dioxide (CO_2_) sink. Recent research has sparked concern that seabed disturbance from trawling can therefore turn marine sediments into a large source of CO_2_, but the calculations involved carry a high degree of uncertainty. This is primarily due to a lack of quantitative understanding of how trawling mixes and resuspends sediments, how it alters bioturbation, bioirrigation, and oxygenation rates, and how these processes translate into carbon fluxes into or out of sediments.

**Methods:**

The primary question addressed by this review protocol is: how does mobile bottom fishing affect benthic carbon processing and storage? This question will be split into the following secondary questions: what is the effect of mobile bottom fishing on: (i) the amount and type of carbon found in benthic sediments; (ii) the magnitude and direction of benthic-pelagic carbon fluxes; (iii) the biogeochemical, biological, and physical parameters that control the fate of benthic carbon; and (iv) the biogeochemical, biological, and physical parameters that control the fate of resuspended carbon. Literature searches will be conducted in Web of Science, SCOPUS, PROQUEST, and a range of grey and specialist sources. An initial scoping search in Web of Science informed the final search string, which has been formulated according to Population Intervention Comparator Outcome (PICO) principles. Eligible studies must contain data concerning a change in a population of interest caused by mobile bottom fishing. Eligible study designs are Before and After, Control and Impact, and Gradient studies. Studies included at full-text screening will be critically appraised, and study findings will be extracted.Extracted data will be stored in an Excel spreadsheet. Results will be reported in narrative and quantitative syntheses using a variety of visual tools including forest plots. Meta-analysis will be conducted where sufficient data exists.

**Supplementary Information:**

The online version contains supplementary material available at 10.1186/s13750-024-00348-z.

## Background

The seafloor covers ~ 71% of the Earth’s surface [1], much of which is covered in marine sediments. These sediments store an estimated 2,322 (2,239–2,391) petagrams (Pg; x 10^15^) of organic carbon in the top 1 m [2], equivalent to almost twice as much carbon as is found in terrestrial soils [3] or the atmosphere (see [12]), making them a globally significant carbon store (i.e., an environment that currently holds a large quantity of carbon) and potential sink (i.e., an environment that takes up atmospheric carbon dioxide (CO_2_)). Some 49% of this stored carbon is located within Exclusive Economic Zones (EEZs), giving individual nations ultimate control over its protection, but only 2% is currently located within areas with sufficient protection as to prevent its disturbance [2].

Mobile bottom fisheries constitute the most widespread physical disturbance to seabed habitats [4], affecting an estimated 4.9 million km^2^ [5]. This is equivalent to ~ 1.5% of the global ocean and 13% of the world’s shelf seas [6], in agreement with an earlier study which reported that 14% of the 7.8 million km^2^ of shelf seas studied had been trawled within the previous 2 years [7]. This disturbance of surface sediments enhances mixing and resuspension and causes shifts in faunal assemblage, each of which may alter rates of organic carbon (OC) remineralisation, OC and inorganic carbon (IC) sedimentation, and consolidation. Bottom fishing also affects benthic organisms, which include bacteria and micro-, meio-, macro- and mega- fauna and flora. These organisms also store carbon within their biomass, estimated at ~ 110 Mt globally [8], and mediate a range of biological, physical, and chemical processes that control local ecosystem properties and, ultimately, influence the rate at which this sedimentary carbon accumulates [9, 10].

A recent global extrapolation suggested that bottom fishing disturbance can turn marine sediments into a large source of CO_2_, releasing an estimated 0.4 Pg C or 1.47 Pg CO_2_ into the water column each year [5]. This number is equivalent to ~ 13% of atmospheric CO_2_ absorbed by the ocean each year [11], ~ 4% of annual anthropogenic CO_2_ emissions [12] or the total annual loss of soil carbon resulting from global agriculture [13]. Ongoing debate as to the veracity of this estimate [14–17] has highlighted a need to better understand and constrain the processes through which mobile bottom fishing might mediate the release of benthic carbon stores as CO_2_, or impede carbon sequestration in sediments by obstructing natural burial processes [18–20]. The overall goal of this review is therefore to synthesise empirical evidence needed to quantify the acute (short-term, resulting from one exposure or trawling event) and chronic (long-term, resulting from multiple and/or long-term exposures or trawling events) effects of bottom fishing on benthic carbon stores.

Many and varied biogeochemical and physical interactions occur following bottom fishing activity, whether within sediments, the water column, or at the sediment-water interface (SWI). The interactive and aggregative net effect of these processes comprises shifts in sediment and water column chemistry, benthic and pelagic biology (including fauna, flora, and microbes), geology (including seabed lithology and granulometry), and local hydrodynamics [21, 22]. Some of these interactions are relatively straightforward, for example a deepening of the oxygen penetration depth (OPD) might alter remineralisation of sedimentary organic carbon. Other interactions can initiate more complex pathways, the results of which are more difficult to predict. For example, a reduction in benthic biomass may decrease the stability of surficial sediments, leading to increased suspended sediment and a range of shifts in water column properties including light attenuation, nutrient concentration, and microbial abundance, each of which may influence organic carbon remineralisation and, by extension, carbon storage. However, the net effect of these complex processes on carbon processing and storage is uncertain [18].

We will therefore examine both the direct and indirect effects of bottom fishing on the biological, chemical, and physical factors which mediate carbon sequestration, remineralisation, and storage. We define carbon sequestration as the collective processes that add carbon to the benthic carbon store. This includes carbon added to sediments or benthic organisms including photosynthetic organisms (e.g. microphytobenthos, kelp, seagrass) and organisms that form calcium carbonate shells (e.g. molluscs). The burial of POC and PIC in sediment is also included within the term carbon sequestration. Carbon sequestration depends upon factors such as particle settlement rate, the relative abundance of organic matter, and the type of organic matter being buried (among other variables). Mobile bottom contact fishing practices may thus impact on carbon sequestration and burial by changing any of these factors. Carbon remineralisation is defined by the collective biogeochemical processes that remove carbon from the benthic carbon store. This includes recycling processes in which carbon such as dead cells or metabolites are decomposed into smaller organics and further degraded to dissolved inorganic carbon (DIC). Remineralisation is primarily driven by microbial activity but is also affected by other biological (e.g. bioturbation and bioirrigation) and physico-chemical (e.g. redox, pH, electron acceptors like oxygen and nitrate) factors. Carbon storage is defined as carbon stored in benthic systems, independent of timescale (seasonal-interannual) or form (dissolved, particulate, living, non-living).

**Fig. 1:**
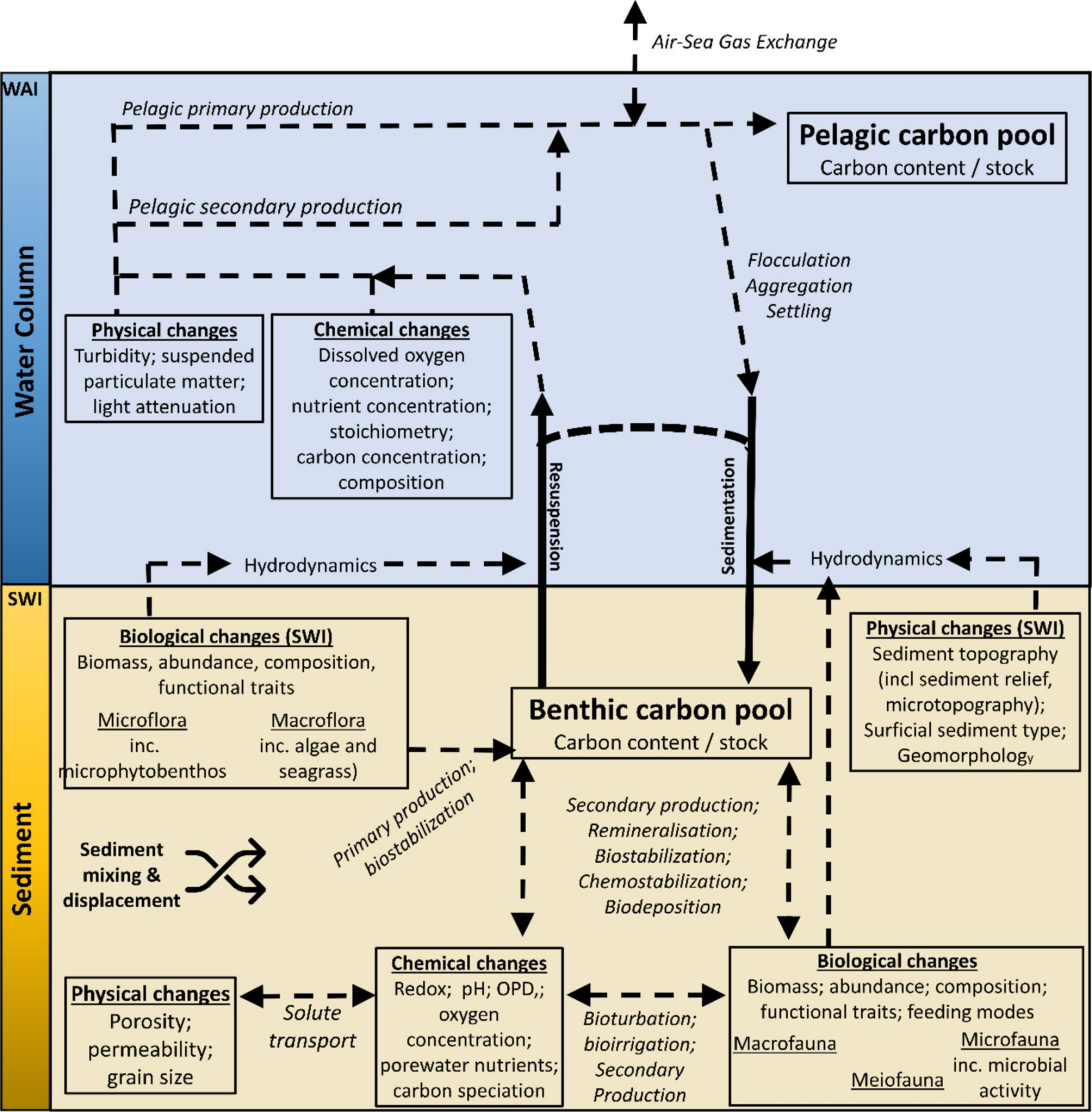
Schematic representation of pathways through which bottom fishing might influence benthic carbon processing and storage. Boxes = outcomes. Arrows = interactions. SWI = Sea Water Interface; WAI = Water Air Interface; OPD = Oxygen Penetration Depth

Mobile bottom fishing is expected to influence these processes in a wide range of ways (Fig. 1; Table S1) which can be broadly grouped according to (a) physical alteration of sediments by bottom fishing gear; (b) physical damage to benthic biota by bottom fishing gear; and (c) resuspension of surface sediments following a trawl pass.

## Objective of the review

This review will assemble a comprehensive, up-to-date database on how mobile bottom fishing affects benthic carbon processing and storage and will be used to address the following primary and secondary questions:

### Primary question

How does mobile bottom fishing affect benthic carbon processing and storage?

### Secondary questions

How does mobile bottom fishing affect.

i) the quantity and type of carbon found in benthic sediments;
ii) the magnitude and direction of benthic-pelagic carbon fluxes;
iii) the geochemical, biological, and physical parameters that control the fate of benthic carbon; and.
iv) the geochemical, biological, and physical parameters that control the fate of resuspended carbon?

The scope of this review will be global, with a focus on subtidal habitats including coastal, shelf, and deep-sea marine areas. Intertidal habitats such as mangroves and saltmarshes are not included.

Moreover, to place the effects of mobile bottom fishing in full context requires better quantification of the effects of different anthropogenic pressures (e.g. channel dredging) on carbon processing and storage and understanding of how these effects compare with natural hydrological disturbances to seabed sediments in contrasting environmental settings. Therefore, we will also extract data that compares the effect of bottom fishing to other anthropogenic and natural disturbances.

The resulting synthesis will provide critical knowledge and understanding required to inform discussions around the role of nature in supporting net-zero ambitions [23] and policy around climate change objectives and marine protected areas as Nature Based Solutions (NBS). It will also identify knowledge gaps, helping to guide future research efforts more effectively. The resulting synthesis could also be used to parameterize and validate the ecosystem and biogeochemical models used to inform local, regional, and global predictions of trawling impacts on benthic carbon budgets (e.g. the European Regional Seas Ecosystem Model (ERSEM; [24] and the OMEXDIA diagenetic modelling framework [25, 26]).

### Methods

#### Stakeholder engagement

An expert Scientific Advisory Panel (SAP) comprised of subject experts in carbon cycling, benthic and pelagic biogeochemistry, benthic biota, and biogeochemical modelling were consulted via an online questionnaire survey (File S2) and during a 2-day hybrid workshop (20th and 21st April 2023).

The questionnaire asked the SAP to identify important processes and/or pathways by which bottom trawling might influence the remineralisation, accumulation, and storage of carbon within benthic environments, including parameters which might be affected and associated literature and datasets. The questionnaire also asked the SAP to suggest other forms of natural or anthropogenic disturbance which might serve as analogues to disturbance caused by bottom trawling.

During the workshop, the SAP considered the questionnaire responses and supplemented them where necessary, before agreeing upon a list of important processes and/or pathways (File S1) and a list of bench-mark articles which were identified as being highly relevant (File S3). The SAP also produced and agreed upon a list of data sources to be searched, including bibliographic databases, search engines, dissertation databases, and specialist websites (see *Planned Searches*). After the workshop, the SAP were consulted via email on search string development, inclusion criteria, and the finished protocol text. All members of the SAP are included as co-authors, and each provided feedback on the document and agreed to its contents.

The SAP will continue to be involved in the process and will be invited to join the article review team and to contribute to the resultant publication(s). No other stakeholder groups are involved.

## Searching for articles

### Search languages

The search will be conducted in English only.

### Search string formulation

We initially built a search string in R (v. 4.3.1; [27] using the ‘LitsearchR’ package [28]. This involved conducting a naïve search in Web of Science:

TS = (carbon OR biogeochem* OR fauna OR flora OR resuspension) AND (benth* OR sediment OR demersal) AND (fishing OR trawling).

This search identified a total of 6,749 results, the title, author, year, keyword, and abstract data for which were exported as a text file. Litsearch R was used to extract specified keywords and any phrases of 2 or more words which occurred more than five times in total, and these were manually checked for relevance. We manually added specific search terms identified by the SAP as being of high importance, categorised all terms as a Population, Intervention, Comparator, or Outcome according to PICO principles [29] and had LitsearchR construct a search string based on that categorisation.

### Search string

(benth* OR *benthic OR *bottom OR demersal* OR sediment* OR seabed)

AND

(bacteria* OR biogeochem* OR biomarker$ OR biot* OR bioturb* OR carbon* OR “chemical characteristic*” OR “chemical composition” OR “chemical parameter*” OR chlorophyll OR “dissolved inorganic” OR “dissolved organic” OR DOC OR *fauna OR fauna* OR *flora OR geochemistry OR habitat OR isotop* OR maerl OR methane OR microb* OR nitrogen* OR nutrient* OR “organic matter*” OR oxygen* OR phosph* OR POC OR “primary consum*” OR “primary produc*” OR resuspen* OR “secondary consum*” OR “secondary produc*” OR sulf* OR sediment* OR sulph* OR “suspended partic*” OR “suspended sediment*” OR “suspended solid*” OR TOC)

AND

(drag* OR dredg* OR harvest* OR trawl* OR *trawl OR fishing OR fisher*)

AND

(abundance OR accumul* OR activit* OR bioavailabil* OR biomass OR composition OR concentration* OR content OR distribution OR *diversity OR erosion OR flux* OR growth OR hydrodynamics* OR metabolism OR *mineralisation OR *mineralization OR quality OR rate* OR ratio* OR respiration OR resuspension OR sedimentation OR signature OR stock OR structure OR turbid*)

The asterisk (*) at the end of a search term/word was used to accept any variant of a base term. The.

dollar ($) was used to accept single or no added characters, useful for acquiring plural and singular forms. Quotation marks were used to search the exact word order. The evolution of this search string is detailed in the additional datafile (S3).

A database on the effects of mobile bottom fishing on benthic biota and their functional traits already exists. This database was generated according to a previously published systematic review protocol [30] and will be used to supplement our search, which does not explicitly target functional trait data. This database was last updated in January 2023.

### Comprehensiveness of final search string

Search comprehensiveness was assessed using the Web of Science Core Collection and 273 bench-mark articles which were identified by the SAP as being highly relevant (File S3). The search string was then manually edited to achieve inclusion of all bench-mark studies. 20 of these benchmark studies could not be returned by the search, the reasons for which are given in File S3.

### Planned searches

We intend to repeat the above search across a wide range of databases to ensure we maximise retrieved data, adapting the search string to each search facility according to the maximum allowable string length and specific search syntax. Any changes made to the search string will be reported in the final review.

All web-based searches will be conducted whilst logged out of online accounts (e.g., Google) and we will delete browsing history and cookies before each search. A record of each search will be kept, and all searches will be conducted from a single university laptop to ensure all software settings / versions are kept consistent. For bibliographic databases, we will consider all hits. For Search Engines, the first 1000 hits will be included. For dissertation databases and grey literature, the first 50 hits will be included. These cut-off values were selected based on initial scoping searches, where 1000 and 50 were more than sufficient to capture the relevant material. In all cases, searches will be sorted by relevance. Review papers will be flagged and their reference sections cross-checked against our final database to identify any additionally relevant papers that may have been missed. Heriot-Watt University access agreements will be used to access all sources held behind paywalls. Where university access does not exist, we will attempt to get copies through the Inter-Library Load Service in the UK. Failing that, we will contact authors directly to request access.

All reviewers agree not to screen, code, judge the validity of, or in any other way handle any studies to which they have contributed or been involved in.

### Bibliographic searches

Web of Science (http://wokinfo.com) – Core Collection.

Scopus (www.scopus.com).

ProQuest (https://www.proquest.com).

### Dissertation searches

Index to Theses Online (www.theses.com).

Digital Dissertations Online (http://www.lib.umn.edu/indexes/digitaldissertations).

### Search engines

Google (www.google.com).

Google Scholar (www.scholar.google.com).

### Specialist searches for grey literature

American Fisheries Society (https://fisheries.org).

Australian Society for Fish Biology (https://www.asfb.org.au).

British Ecological Society (https://www.britishecologicalsociety.org).

Centre for Environment, Fisheries, and Aquaculture (https://www.cefas.co.uk).

Commonwealth Scientific and Industrial Research Organisation (https://www.csiro.au).

Department for the Environment, Food, and Rural Affairs (https://www.gov.uk/government/organisations/department-for-environment-food-rural-affairs).

Department of Fisheries and Oceans Canada (https://www.dfo-mpo.gc.ca/index-eng.htm).

Esme Fairburn Association (https://esmeefairbairn.org.uk/open-data).

Food and Agriculture Organisation (http://www.fao.org/home/en).

French Research Institute for the Exploitation of the Sea (https://tethys.pnnl.gov/organization/frenchresearch-institute-exploitation-sea-ifremer).

International Council for the Exploration of the Sea (https://www.ices.dk/data/Pages/default.aspx).

International Seafood Sustainability Foundation (https://iss-foundation.org/who-we-are/about).

Japanese Society of Fisheries Science (https://jsfs.jp/en).

Joint Nature Conservation Committee (https://jncc.gov.uk).

Korean Society of Fisheries and Aquatic Sciences (http://www.kosfas.or.kr/main_en.php).

Marine Conservation Alliance (http://marineconservationalliance.org).

Marine Fish Conservation Network (https://conservefsh.org).

Marine Life Information Network (https://www.marlin.ac.uk).

Marine Scotland (https://www.gov.scot/marine-and-fisheries).

Marine Stewardship Council (https://www.msc.org/home).

National Environment Research Council (https://nerc.ukri.org).

National Institute of Water and Atmospheric Research (https://www.niwa.nz).

National Oceanic and Atmospheric Administration (https://www.noaa.gov).

Natural England (https://www.gov.uk/government/publications/natural-englands-publications-mapsand-data).

Natural Resources Wales (www.naturalresourceswales.gov.uk).

North Pacific Marine Science Organization (https://meetings.pices.int).

Northern Ireland Environmental Agency (https://www.daera-ni.gov.uk/northern-ireland-environment-agency).

Northwest Atlantic Fisheries Organisation (https://www.nafo.int).

Oceana (https://oceana.org).

Pew Trusts (https://www.pewtrusts.org/en).

Royal Netherlands Institute of Sea Research (https://www.nioz.nl/en/research).

Scottish Association for Marine Science (https://www.sams.ac.uk).

Scottish Environmental Protection Agency (https://www.sepa.org.uk).

Scottish Natural Heritage - Nature Scot (https://www.nature.scot).

Seafish (https://www.seafish.org/article/selling-directly-to-consumers).

Sustainable Fisheries Partnership (https://www.sustainablefsh.org).

The Nature Conservancy (https://www.nature.org/enus).

Worldwide Fund for Nature (https://www.wwf.org.uk).

### Search record database

All articles and documents will be exported into separate collections using the reference management software Endnote. After all searches have been carried out, references from each search will be merged into one database. Duplicates will then be identified and removed. This database will be uploaded to an online archiving platform (SysRev) and made freely available at the end of the project.

### Article screening and study eligibility criteria

#### Screening process

Screening will be performed at title, abstract, and then full-text levels using the SysRev review management platform. Reviewers agree not to screen any studies they have co-authored or been involved in. For any studies excluded at full-text stage, we will record the reason for exclusion and make this available along with the final database.

### Consistency checking

To ensure consistency and accuracy throughout the screening process amongst the members of the review team, a random subset of 10% of publications will be independently screened by all reviewers. Results will be analysed using Cohen’s Kappa test (Cohen 1960) and used to ascertain the level of agreement amongst the reviewers. If the initial results show ‘substantial’ (K = 0.61–0.8) or ‘almost perfect’ agreement (K = 0.81-1.0), the reviewers will not receive further training, but disagreements will be discussed nonetheless to ensure the best possible outcomes are achieved. If the Kappa score is less than 0.61, disagreements will be discussed and further training will be provided to resolve the issues, and Kappa scores will be calculated for another subset of 10% of publications. This process will be repeated until the Kappa score is greater than 0.61.

#### Study eligibility criteria

Whilst our primary interest is in benthic carbon, population and outcome terms that relate to associated biogeochemical and biological parameters and processes which are known to mediate carbon processing and storage (Fig. 1) will also be eligible for inclusion.

##### Eligible populations

Studies providing data concerning organic and inorganic carbon in total (TOC, TIC), particulate (POC, PIC), and dissolved (DOC, DIC) form and on sediment biogeochemical parameters that mediate carbon storage and processing (e.g. nutrients (nitrogen, phosphorous, silicate), metals and oxygen in the sediment). Studies providing data on changes in resuspended and in-situ sediment characteristics (e.g. grain size, porosity), and changes in biota including microbes, bacteria, macrofauna, meiofauna and micro- and macro-flora present in the sediment. We will only consider studies which include an interaction with the benthic environment (e.g. ‘benthic’, ‘bottom’, ‘demersal’).

##### Eligible interventions

Exposure to mobile bottom fishing gear (e.g. ‘trawl’, ‘dredge’). Table 1 provides a full list of accepted fishing gears.

**Table 1:** Types of bottom fishing gear included in this review (based on FAO classification of fishing gears [31]

Gear type	Description
Beam trawl	A trawl that is towed on the seabed where the net is held open by a beam. This type of trawl targets species which live on top of the sediment (e.g. demersal fish species).
Otter trawl	A trawl that is towed on the seabed where the net is held open by ‘trawl doors’ or ‘otter boards’. This type of trawl targets species which live on top of the sediment (e.g. demersal fish species).
Towed dredge	A trawl with a metal dredge rigged with a scraper blade or teeth along the lower leading edge. This type of trawl targets species which live within the upper sediment (e.g. scallops).
Mechanized dredge	A trawl which uses pressurised water jets to fluidize surface sediments and dislodge target species. This type of trawl targets species which live within the upper sediment (e.g. clams). Also known as a hydraulic dredge.
Boat Seine	A cone-shaped net with elongated wings, seine ropes, and a codend which is used on a smooth seabed. Compared to a trawl net, a seine net usually has longer wings and utilizes heavy ropes that extend from the wings of the net to increase the area over which fish are herded. Also known as ‘fly shooting’, ‘fly dragging’, demersal seine, or a Danish Seine.

##### Eligible comparators

Areas with no demersal trawling, areas with low levels of demersal trawling, trawling gradient studies, areas fished with static gear.

*Eligible Outcomes*: For carbon, nutrient, chlorophyll, and metal populations, the main outcomes of interest will be ‘content’, ‘concentration’, ‘stock’, ‘flux’, and ‘rate’. We will also search for data on carbon ‘accumulation’, ‘composition’, ‘content’, ‘bioavailability’, ‘lability’, ‘metabolism’, ‘re-mineralisation’, ‘quality’, and ‘reactivity’. For carbon isotope populations, the outcomes of interest will be ‘signature’ and ‘ratio’. Outcomes relating to sediments will be ‘erosion’, ‘sedimentation’, and ‘turbidity’. We will also include a ‘hydrodynamics’ term. The following outcomes will be accepted for biological populations: ‘abundance’, ‘density’, ‘activity’, ‘biomass’, ‘composition’, ‘distribution’, ‘diversity’, ‘growth’, ‘respiration’, and ‘structure’.

##### Eligible types of Study Design

We will accept Before and After (BA) studies, Control and Impact (CI) studies, and combinations of the two (BACI). We will also accept gradient studies that compare at least two sites experiencing different levels of exposure. We will accept both field and laboratory studies. Modelling studies are excluded.

We define a study as a discrete experimental or comparative unit, where data from a single source (e.g., a research paper or database) was collected under different environmental conditions (e.g., depth, sediment type, season), from different geographical locations, using different intervention types, or under different levels of fishing intensity (unless a gradient study).

### Study validity assessment

.

A critical appraisal of studies that pass the full-text screening stage will be undertaken for the purposes of excluding unclear studies that suffer from reporting bias (“unclear validity”), and for assessing the impact of studies with low internal and external validity relative to those with medium and high validity on the reliability of the evidence base (“low” vs. “medium” vs. “high” validity).

A study will be categorised as “unclear validity” if:

- the methodological description is poor as a result of key information about study design (column K in File S4 Quantitative tab) and intervention are missing (column S in File S4 Meta tab),
- replication is not adequate - i.e. less than two independent experimental/observational units (columns U, X, AA, AD in File 4 Quantitative tab).

A study will be classified as “low validity” if:

- intervention (i.e. fished) and comparator sites are not matched (e.g. different habitat or ecosystem type), or there are confounding factors present (e.g. additional treatments carried out at the fished sites but not at the comparator sites, or only before or only after the modification) (column L in File 4 Quantitative tab),
- different sampling methodology were used to collect data from intervention and control sites (column N in File S4 Quantitative tab).

A study not assessed to have “low” or “unclear” validity will be considered to have “medium validity” if study:

- design consists of Before-After (BA) (i.e. multiple temporal observations of a single unit in one study context) or Control-Impact study design (column K in File S4 Quantitative tab),
- replication of sample lacks true independence between observational units (pseudo-replication), or unbalanced sampling design (column M in File S4 Quantitative tab).

A study not assessed to have “low”, “medium” or “unclear” validity will be considered to have “high validity” if study:

- design consists of Before-After-Control-Impact (BACI) (column K in File S4 Quantitative tab).

Studies deemed to be “unclear” will be excluded from quantitative synthesis but will still be included in the narrative synthesis. Reasons for exclusion will be recorded for all studies. Studies with “high”, “medium” and “low” validity will be included in the narrative and quantitative synthesis, and the validity rating will be used as a basis for sensitivity analysis in the meta-analyses. The purpose of retaining less reliable studies and performing a sensitivity analysis is to investigate whether such studies are likely to show results that conflict with those from more reliable studies.

The validity of a study will be appraised by two reviewers. A small subset of the studies (10%) will be appraised by both reviewers at the beginning of the appraisal to check for appraisal consistency, and all disagreements will be discussed, and the criteria further refined if deemed necessary.

#### Data coding and extraction strategy

We will extract sample sizes, means, and measures of variation (e.g. confidence intervals, standard errors, and standard deviations), with summary statistics generated from raw data when these are not already provided in the article. Where data are presented in graph form only, we will use software (e.g., PlotDigitizer) to manually extract these data, and will flag this in the final database. We will also extract data on study design, intervention type, frequency, and duration, habitat type, and sediment type. Where harmonization of classifications is required (i.e. for habitat and sediment type), categorisation will be discussed with the SAP and made explicit in the resultant review. The specific metadata on study design and effect modifiers to be extracted are described in our data coding sheet (File S4).

The data extraction process will be carried out by one author, with independent testing conducted on a sub-set of studies by a second author (10%). A sub-set of papers will be double extracted by both authors and agreement reached before moving forward. Additional checks will be conducted throughout the extraction process to ensure agreement is consistent throughout the process. If any disagreement or uncertainty arises, the wider team will be consulted until agreement is reached.

Where raw data, measures of variation, or metadata are missing or unclear, the corresponding author will be contacted in the first instance to request clarification. Where the corresponding author is not responsive, we will reach out to additional co-authors.

All extracted data will be stored in an excel database which will be provided as a supplementary file alongside final results.

#### Potential effect modifiers

The degree of mobile bottom fishing disturbance is both context- and site- specific. For instance, both the size and longevity of benthic fauna [33] and the penetration depth of the fishing gear used [34] have been shown to influence the magnitude of benthic disturbance on seabed fauna and biogeochemistry. The following list contains potential effect modifiers identified by the Scientific Advisory Panel. Data on these effect modifiers will be recorded in an Excel spreadsheet (File S4), alongside any additional effect modifiers identified during the extraction process. All effect modifiers will be coded and included in subsequent analysis.

- Benthic habitat type.
- Depth of sampling site.
- Environmental factors (e.g., water temperature, currents, water column stratification, salinity, dissolved oxygen in bottom water).
- Geographical factors (e.g., reef, mound, front).
- Fishing intensity.
- Haul frequency.
- Gear design.
- Gear type.
- Geographical coordinates.
- Historical fishing pressure.
- Exposure duration.
- Seasonality (including pre- and post-algal bloom).
- Study duration.
- Study sample size.
- Time interval(s) between impact and sampling.
- Sampling technique.
- Sediment depth slice for measurements taken throughout the sediment depth profile.
- Baseline sedimentation rate of study site / area/ region.
- Particulate organic carbon flux to the seafloor.

#### Data synthesis and presentation

Once assembled, this database will be used to (a) identify existing knowledge clusters and gaps with regards to the effect of mobile bottom fishing on benthic carbon processing and storage; and (b) address the primary and secondary questions outlined above (see *Objective of the review*).

Knowledge gaps will be identified using heatmaps, which will be constructed by cross tabulating data on key descriptors. We will present metadata descriptively, grouping studies according to e.g., geographic location, intervention type, and parameter measured and presenting a descriptive analysis through tables, figures, and a narrative description of the evidence base.

Where quantitative data is available, we will conduct a quantitative analysis. In this instance, data from eligible studies with comparable effect sizes will be standardized and weighted appropriately. Where sufficient quantitative data exists, meta-analysis will be used to assess the effect of demersal trawling on each identified population and outcome combination. This meta-analysis will be conducted in R using the *metafor* package [35] focusing on meta-regression analysis to identify modifiers with the greatest effect. Effect sizes will be determined using for example the natural log transformed response ratio or other appropriate effect size. We will conduct a sensitivity analysis to test the robustness of our validity assessment, and use funnel plots, Egger tests, and comparisons of peer-reviewed and grey literature to assess publication bias. Data synthesis and presentation will be conducted by an author who has not published in this research area, which will limit potential presentation bias. All quantitative data will be presented alongside a description of the validity of our results and a record of study design, outcome measures, and other key descriptors using descriptive plots and tables.

## Electronic supplementary material

Below is the link to the electronic supplementary material.

Supplementary Material 1

Supplementary Material 2

Supplementary Material 3

Supplementary Material 4

## Data Availability

Additional data sharing is not applicable to this article as no datasets were generated or analysed during the current study.

## References

[1] Watling L, Guinotte J, Clark MR, Smith CR. A proposed biogeography of the deep ocean floor. Prog Oceanogr. 2013;111:91–112.

[2] Atwood TB, Witt A, Mayorga J, Hammill E, Sala E. vol 7, 165,. Global Patterns in Marine Sediment Carbon Stocks (2020). Frontiers in Marine Science. 2021;8.

[3] Kochy M, Hiederer R, Freibauer A. Global distribution of soil organic carbon - part 1: masses and frequency distributions of SOC stocks for the tropics, permafrost regions, wetlands, and the world. Soil-Germany. 2015;1(1):351–65.

[4] Hiddink JG, Jennings S, Sciberras M, Szostek CL, Hughes KM, Ellis N, et al. Global analysis of depletion and recovery of seabed biota after bottom trawling disturbance. Proc Natl Acad Sci. 2017;114(31):8301–6. 28716926 10.1073/pnas.1618858114 PMC5547586

[5] Sala E, Mayorga J, Bradley D, Cabral RB, Atwood TB, Auber A, et al. Protecting the global ocean for biodiversity, food and climate. Nature. 2021;592:7854. 10.1038/s41586-021-03371-z 33731930

[6] Longhurst A, Sathyendranath S, Platt T, Caverhill C. An estimate of global primary production in the ocean from satellite radiometer data. J Plankton Res. 1995;17(6):1245–71.

[7] Amoroso RO, Pitcher CR, Rijnsdorp AD, McConnaughey RA, Parma AM, Suuronen P, et al. Bottom trawl fishing footprints on the world’s continental shelves. Proc Natl Acad Sci U S A. 2018;115(43):E10275–82. 30297399 10.1073/pnas.1802379115 PMC6205437

[8] Wei CL, Rowe GT, Escobar-Briones E, Boetius A, Soltwedel T, Caley MJ et al. Global patterns and predictions of Seafloor Biomass using Random forests. PLoS ONE. 2010;5(12). 10.1371/journal.pone.0015323 PMC3012679 21209928

[9] Bianchi TS, Brown CJ, Snelgrove PVR, Stanley RRE, Cote D, Morris C. Benthic invertebrates on the move: a tale of ocean warming and sediment Carbon Storage. Limnol Oceanogr Bull. 2023;32(1):1–5.

[10] Bianchi D, Carozza DA, Galbraith ED, Guiet J, DeVries T. Estimating global biomass and biogeochemical cycling of marine fish with and without fishing. Sci Adv. 2021;7(41):eabd7554. 34623923 10.1126/sciadv.abd7554 PMC8500507

[11] Gruber N, Clement D, Carter BR, Feely RA, van Heuven S, Hoppema M, et al. The oceanic sink for anthropogenic CO2 from 1994 to 2007. Science. 2019;363(6432):1193–. 30872519 10.1126/science.aau5153

[12] Friedlingstein P, O’Sullivan M, Jones MW, Andrew RM, Gregor L, Hauck J, et al. Global Carbon Budget 2022. Earth Syst Sci Data. 2022;14(11):4811–900.

[13] Lal R. Soil carbon sequestration impacts on global climate change and food security. Science. 2004;304(5677):1623–7. 15192216 10.1126/science.1097396

[14] Sala E, Mayorga J, Bradley D, Cabral RB, Atwood TB, Auber A, et al. Reply to: a path forward for analysing the impacts of marine protected areas. Nature. 2022;607(7917):E3–4. 35794263 10.1038/s41586-022-04776-0

[15] Atwood TB, Sala E, Mayorga J, Bradley D, Cabral RB, Auber A, et al. Reply to: quantifying the carbon benefits of ending bottom trawling. Nature. 2023;617(7960):E3–5. 37165243 10.1038/s41586-023-06015-6

[16] Hiddink JG, Van De Velde SJ, McConnaughey RA, De Borger E, Tiano J, Kaiser MJ, et al. Quantifying the carbon benefits of ending bottom trawling. Nature. 2023;617(7960):E1–2. 37165247 10.1038/s41586-023-06014-7

[17] Hilborn R, Kaiser MJ. A path forward for analysing the impacts of marine protected areas. Nature. 2022;607(7917):E1–2. 35794262 10.1038/s41586-022-04775-1

[18] Epstein G, Middelburg JJ, Hawkins JP, Norris CR, Roberts CM. The impact of mobile demersal fishing on carbon storage in seabed sediments. Glob Change Biol. 2022;28(9):2875–94. 10.1111/gcb.16105 PMC9307015 35174577

[19] Epstein G, Roberts CM. Does biodiversity-focused protection of the seabed deliver carbon benefits? A UK case study. Conserv Lett. 2022:9.

[20] Graves CA, Benson L, Aldridge J, Austin WE, Dal Molin F, Fonseca VG, et al. Sedimentary carbon on the continental shelf: emerging capabilities and research priorities for Blue Carbon. Front Mar Sci. 2022;9:926215.

[21] Middelburg JJ. Reviews and syntheses: to the bottom of carbon processing at the seafloor. Biogeosciences. 2018;15(2):413–27.

[22] Burdige DJ. Preservation of organic matter in marine sediments: controls, mechanisms, and an imbalance in sediment organic carbon budgets? Chem Rev. 2007;107(2):467–85. 17249736 10.1021/cr050347q

[23] Parker R, Benson L, Graves C, Kröger S, Vieira R. Blue carbon stocks and accumulation analysis for Secretary of State (SoS) region. Cefas Project Report for Defra. 2022.

[24] Butenschön M, Clark J, Aldridge JN, Allen JI, Artioli Y, Blackford J, et al. ERSEM 15.06: a generic model for marine biogeochemistry and the ecosystem dynamics of the lower trophic levels. Geosci Model Dev. 2016;9(4):1293–339.

[25] De Borger E, Braeckman U, Soetaert K. Rapid organic matter cycling in North Sea sediments. Cont Shelf Res. 2021;214:104327.

[26] Soetaert K, Herman PM, Middelburg JJ. A model of early diagenetic processes from the shelf to abyssal depths. Geochim Cosmochim Acta. 1996;60(6):1019–40.

[27] Core Team R. R. R: a language and environment for statistical computing. Vienna, Austria: R Foundation for Statistical Computing; 2023.

[28] Grames EM, Stillman AN, Tingley MW, Elphick CS. An automated approach to identifying search terms for systematic reviews using keyword co-occurrence networks. Methods Ecol Evol. 2019;10(10):1645–54.

[29] Booth A, Noyes J, Flemming K, Moore G, Tunçalp Ö, Shakibazadeh E. Formulating questions to explore complex interventions within qualitative evidence synthesis. BMJ Global Health. 2019;4(Suppl 1):e001107. 10.1136/bmjgh-2018-001107 PMC6350737 30775019

[30] Hughes KM, Kaiser MJ, Jennings S, McConnaughey RA, Pitcher R, Hilborn R, et al. Investigating the effects of mobile bottom fishing on benthic biota: a systematic review protocol. Environ Evid. 2014;3(1):23. 10.1186/s13750-024-00348-z PMC11476316 39402692

[31] He P, Chopin F, Suuronen P, Ferro RS, Lansley J. Classification and illustrated definition of fishing gears. FAO Fisheries Aquaculture Tech Paper. 2021(672):I–94.

[32] Konno K, Livoreil B, Pullin AS. Collab Environ Evid Crit Appraisal Tool. 2021;0:3.

[33] Hiddink JG, Jennings S, Sciberras M, Bolam SG, Cambiè G, McConnaughey RA, et al. Assessing bottom trawling impacts based on the longevity of benthic invertebrates. J Appl Ecol. 2019;56(5):1075–84.

[34] Hiddink Jan G, Jennings S, Sciberras M, Szostek Claire L, Hughes Kathryn M, Ellis N et al. Global analysis of depletion and recovery of seabed biota after bottom trawling disturbance. Proceedings of the National Academy of Sciences. 2017;114(31):8301-6. 10.1073/pnas.1618858114 PMC5547586 28716926

[35] Viechtbauer W. Conducting Meta-analyses in R with the metafor Package. J Stat Softw. 2010;36(3):1–48.

