# Supplementary material for: Investigating the effects of mobile bottom fishing on benthic carbon processing and storage: a systematic review protocol
Source: Environ Evid. 2024 Oct 15;13:24. doi: 10.1186/s13750-024-00348-z (PMC11476316; doi:10.1186/s13750-024-00348-z)
Supplement: Supplementary file 1 — Supplementary Material 1 [file 13750_2024_348_MOESM1_ESM.docx]

| **Pathways through which mobile bottom fishing affects carbon storage** | | |
| --- | --- | --- |
|  | **Pathway name** | **Pathway detail** |
| BIOLOGICAL CHANGES | PELAGIC PRIMARY PRODUCTION | Changes in primary production rate in overlying water column due to changes in turbidity, oxygen, and nutrients in water column, which in turn might influence how much particulate organic carbon (POC) reaches the seabed and subsequent release of dissolved organic carbon (DOC) into water column. Nutrient ratio shifts linked to type of phytoplankton. |
|  | PELAGIC SECONDARY PRODUCTION | Changes in secondary production in overlying water column might be affected through changes in pelagic primary production, which in turn might influence how much POC and DOC reaches the seabed, and its composition. |
|  | BENTHIC PRIMARY PRODUCTION | Alteration in the amount and/or composition of benthic flora (photosynthetic organisms e.g., kelp, seagrass, calcareous algae, corals, rhodoliths, microphytobenthos, macroalgae) which might alter carbon (and other nutrients such as nitrogen) sequestration and/or remineralisation. Nutrient ratio shifts linked to type of benthic photosynthetic organism. |
|  | BENTHIC SECONDARY PRODUCTION | Alteration in the amount and/or composition of benthic fauna (macro and meio) which might alter carbon sequestration and/or remineralisation. |
|  |  | Changes in individual body-mass and/or density might cause a change of metabolic rate (respiration). |
|  | BIOTURBATION / BIOIRRIGATION | Changes in the abundance and/or community composition of tube-builders, bioturbators, bioirrigators that move more reactive and refractory carbon away from the oxygen zone, and vice versa. |
|  |  | Changes in the abundance and/or community composition of tube-builders, bioturbators, bioirrigators might alter oxygen and redox conditions of sediment. |
|  | FAUNAL BIOMASS AND TROPHIC CASCADE | Changes in carbon in biological tissue across various trophic levels (organic and inorganic). |
|  | BIODEPOSITION / FEEDING MODES | Changes in the abundance or composition of functional groups (e.g. filter feeders / suspension feeders, deposit feeders) might change the direction and magnitude of water-sediment carbon exchange |
|  |  | Changes in the abundance or composition of reef-building species might alter hydrodynamics with consequences for local and regional carbon deposition and resuspension rates. Species specific effects could mean complete changes in some reef building communities. |
|  |  | Changes in the abundance of benthic foraminifera and macrofauna that form carbonate shells. |
|  | MICROBIAL COMMUNITIES | Changes in community structure, activity of microbiota / microbes / aerobic / anaerobic bacteria / archaea / fungi might lead to changes in aerobic / anaerobic pathways as well as changes in functional traits of microbes. |
| PHYSICAL CHANGES | INHIBITION OF SEDIMENT ACCUMULATION THROUGH RESUSPENSION | Changes in geomorphology, microtopography or seabed relief or surficial sediment type (in terms of particle grain size) might change the overlaying hydrodynamic and settlement rates of... |
|  |  | Changes in accumulation and deposition of… due to erosion and suspension of sediment from trawling. (Net effect will depend on local hydrodynamics). |
|  |  | Loss of finer particles (sediment or particulate carbon) during resuspension will reduce sequestration of organic matter (OM) at the trawl site. (Effect known as winnowing). |
|  | ALTERATION TO SEDIMENT STRUCTURE | Changes to sediment composition, particle grain size, porosity and permeability across the sediment profile (I.e. vertical distribution) caused by winnowing and resuspension of sediment. |
|  | WATER COLUMN TURBIDITY | Change in seawater turbidity through sediment resuspension following trawling may prevent light from reaching the seafloor, which in turn limits benthic primary production which harbours organic carbon. |
| CHEMICAL CHANGES | CHANGES IN CARBON STOCKS & REACTIVITY | Changes in the stocks of POC, particulate inorganic carbon (PIC), dissolved inorganic carbon (DIC), DOC within the sediment depth profile (i.e. carbon relocation with sediment depth profile) due to sediment mixing and resuspension. |
|  |  | Changes in POC and DOC reactivity within the sediment depth profile due to sediment mixing and priming which arises from mixing of labile and refractory carbon pools. |
|  | ALTERATION OF REDOX REGIME IN SEDIMENT | Changes in the redox regime due to physical mixing of the sediment, which influences microbial driven remineralization processes. |
|  | ALTERATION OF ELECTRON ACCEPTORS IN THE SEDIMENT | Changes in electron acceptors (such as oxygen, nitrate) in the sediment (e.g. oxygen penetration depth and oxygen profile) that regulate remineralization reactions through aerobic and anaerobic respiration. |
|  | CHANGES IN BOTTOM WATER OXYGEN CONCENTRATION | Changes in bottom water oxygen and redox concentration as a result of faster remineralization rate of organic matter in response to exposure to oxygen. |
|  | MINERAL INTERACTION | Changes in clay content and e.g., Fe-OC interactions could affect carbon storage. Inclusion of gravel and sands, silt content, particle size fractionation, density, etc |
|  | DISRUPTION OF BIOSTABILISATION | Changes in biology affects sediment stability due to removal of polysaccharides (e.g. microphytobenthos, worm mucal tubes, dead detrital material) |
