# Supplementary figures and images for: Investigating the effects of mobile bottom fishing on benthic carbon processing and storage: a systematic review protocol
Source: Environ Evid. 2024 Oct 15;13:24. doi: 10.1186/s13750-024-00348-z (PMC11476316; doi:10.1186/s13750-024-00348-z)

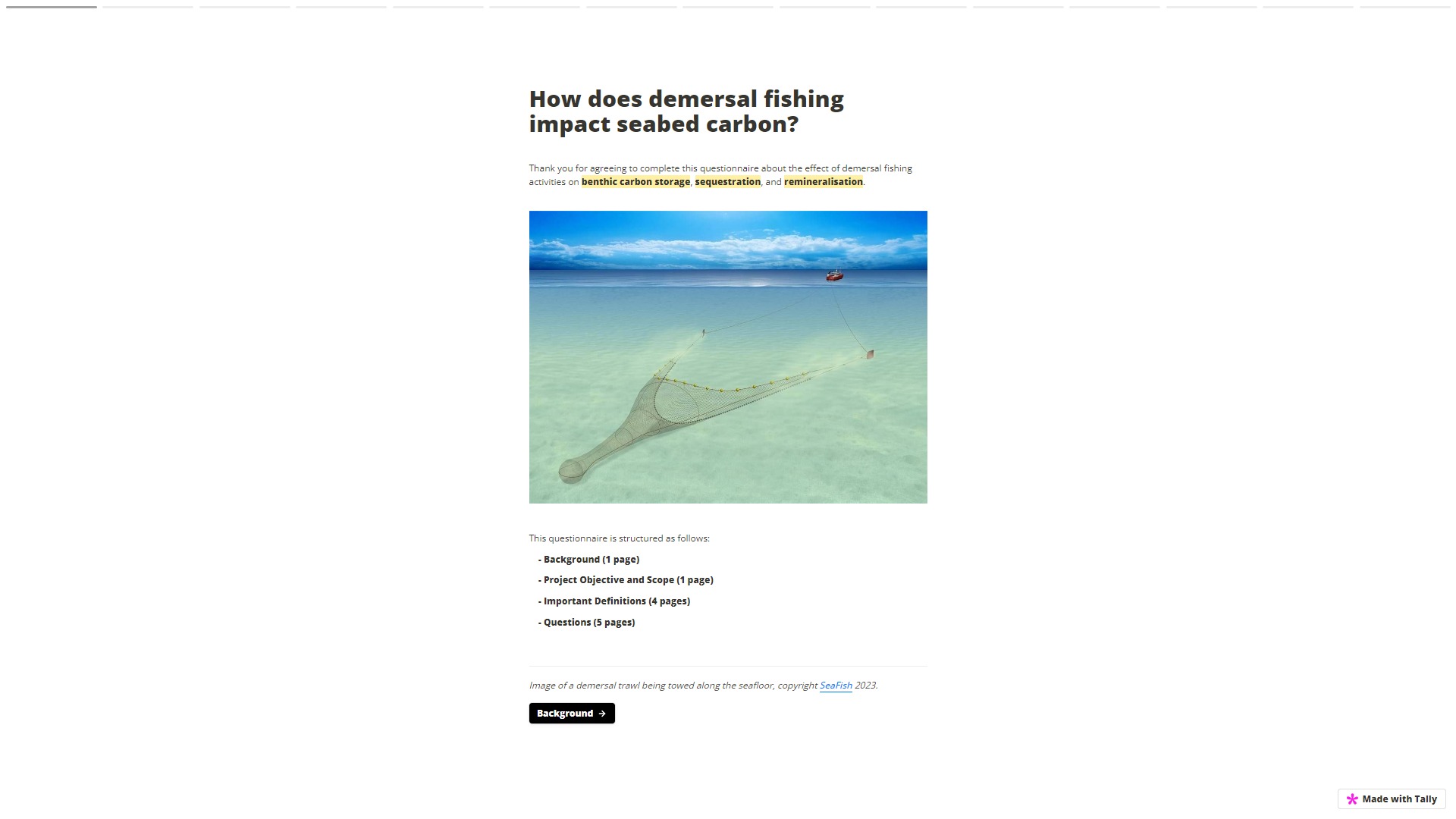


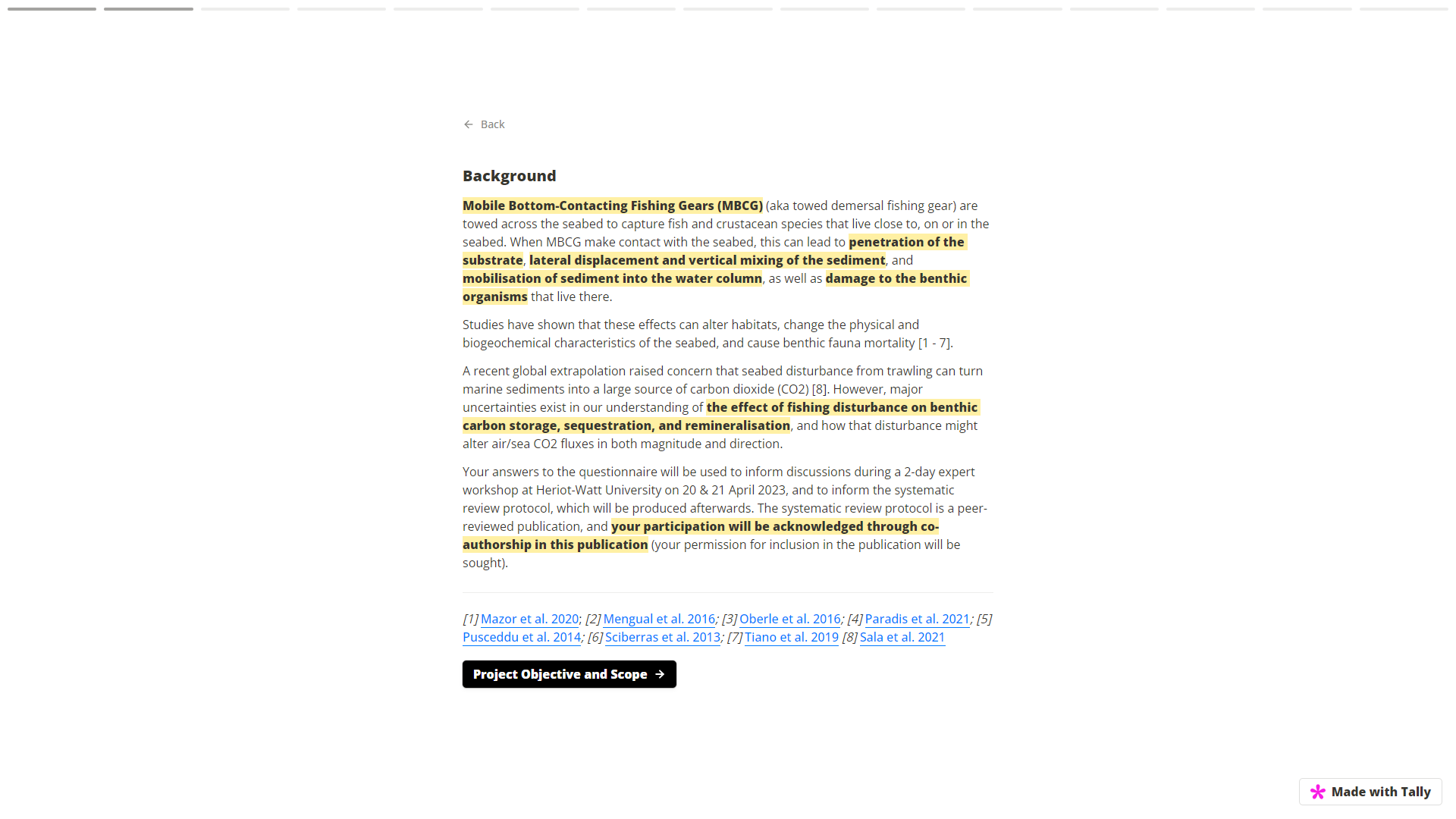


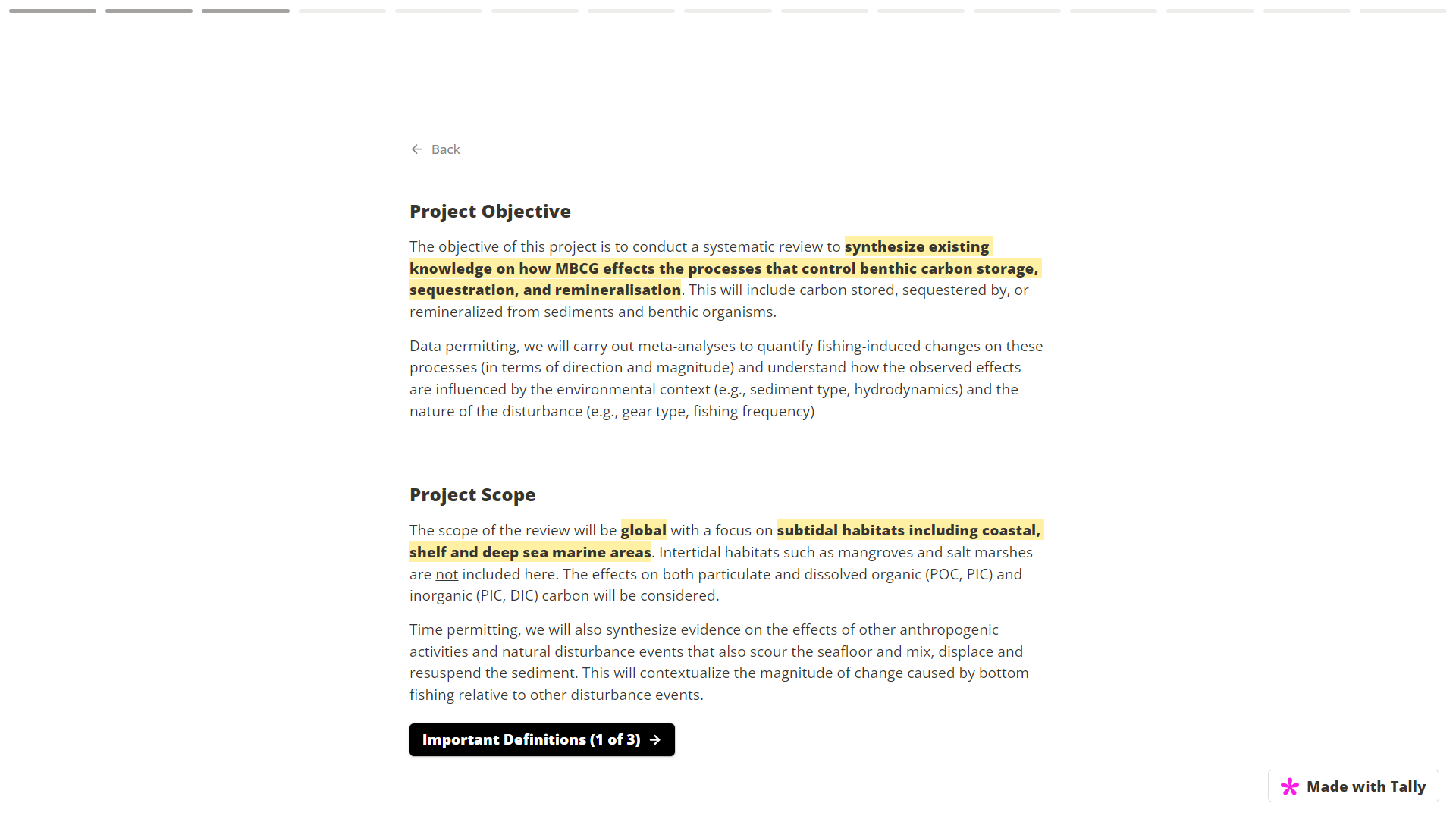


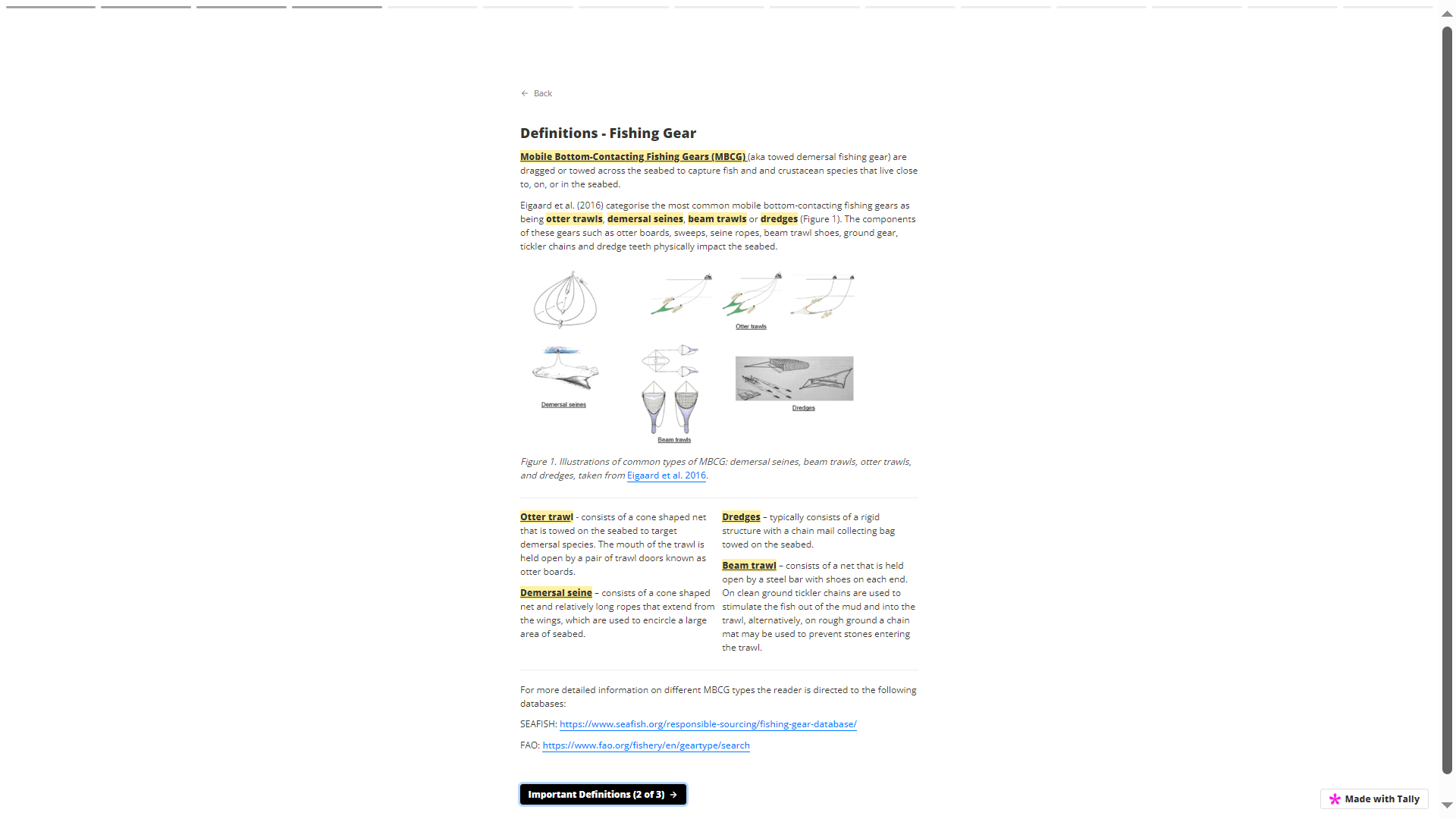


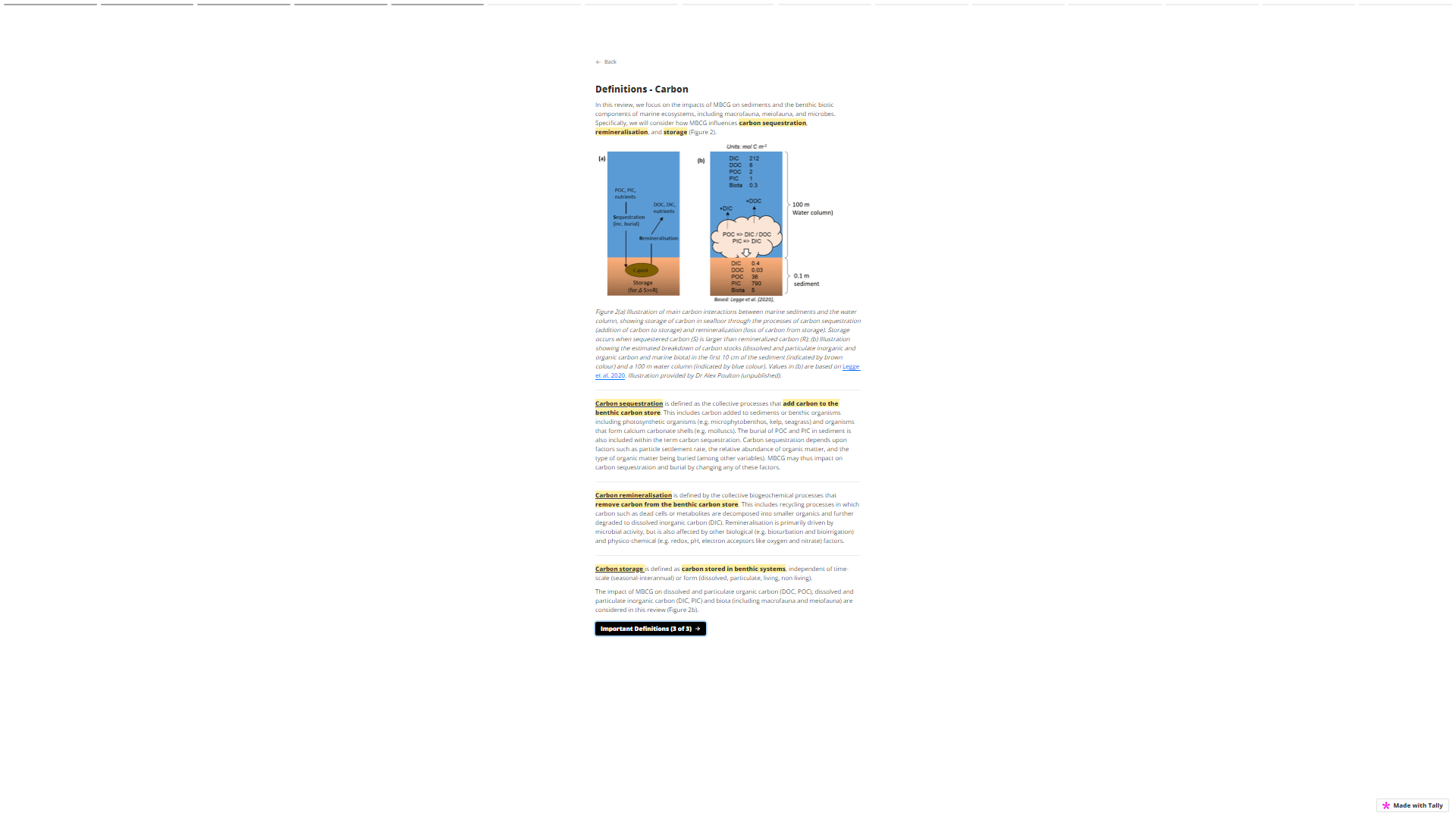


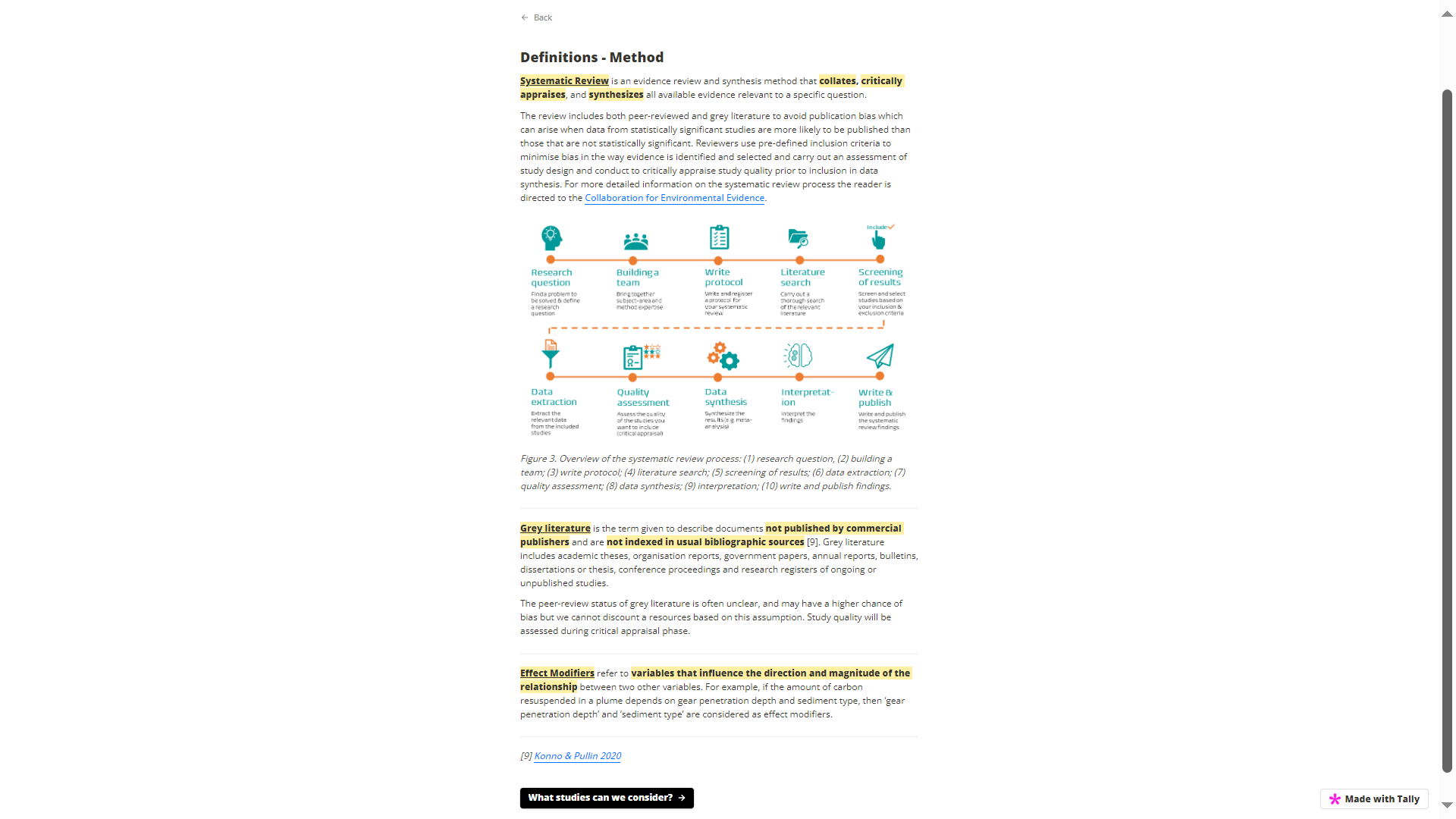


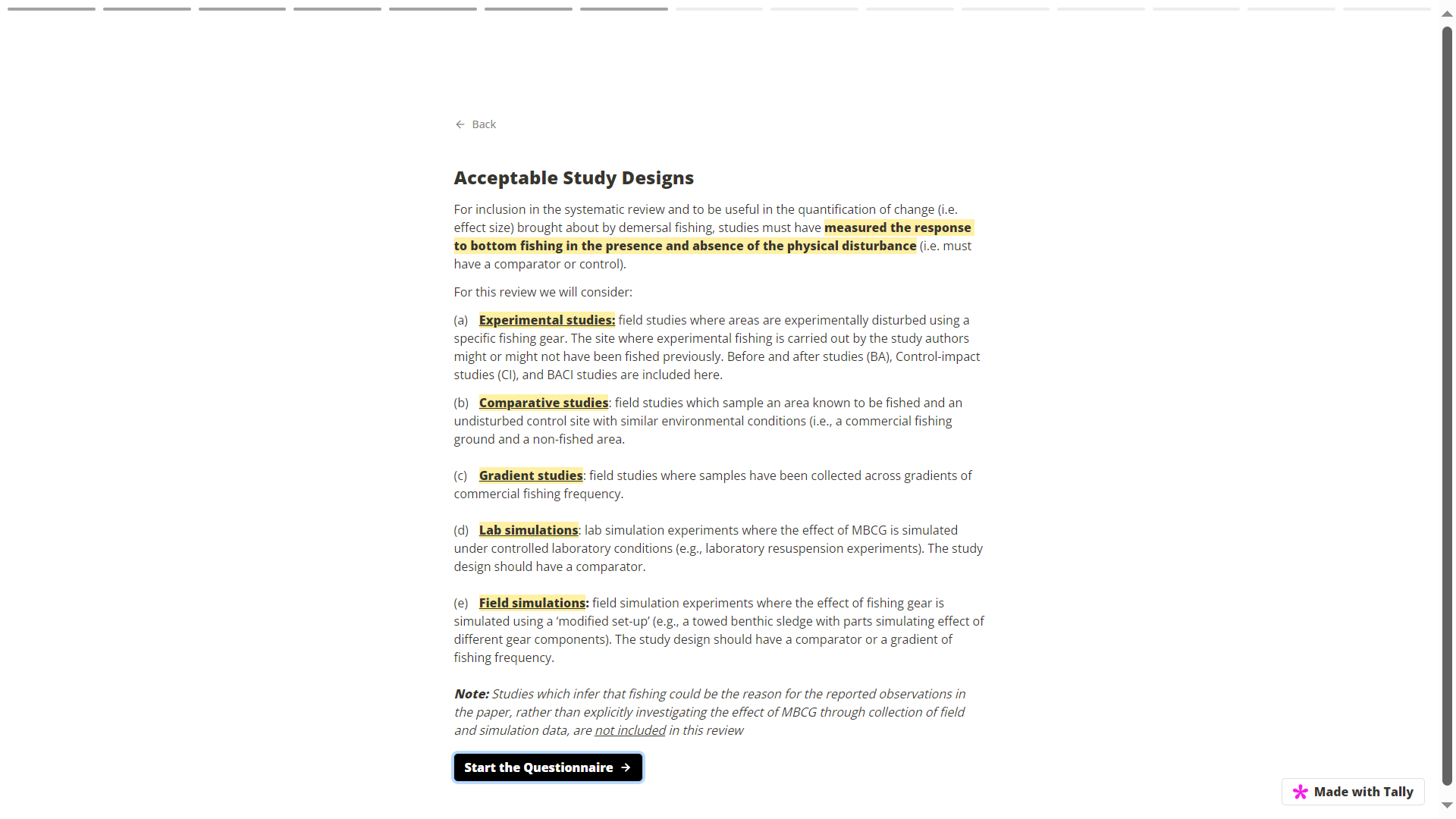


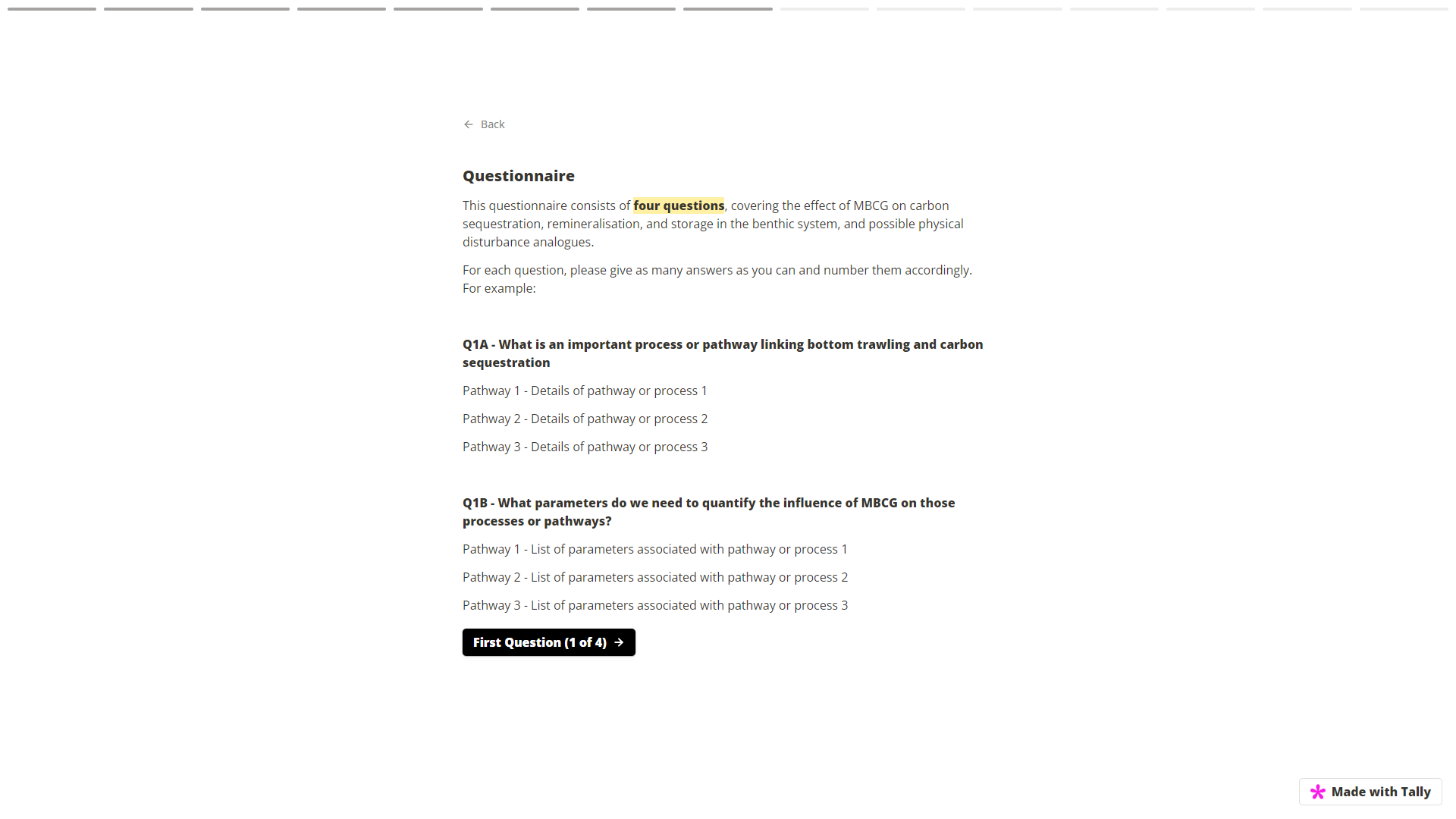


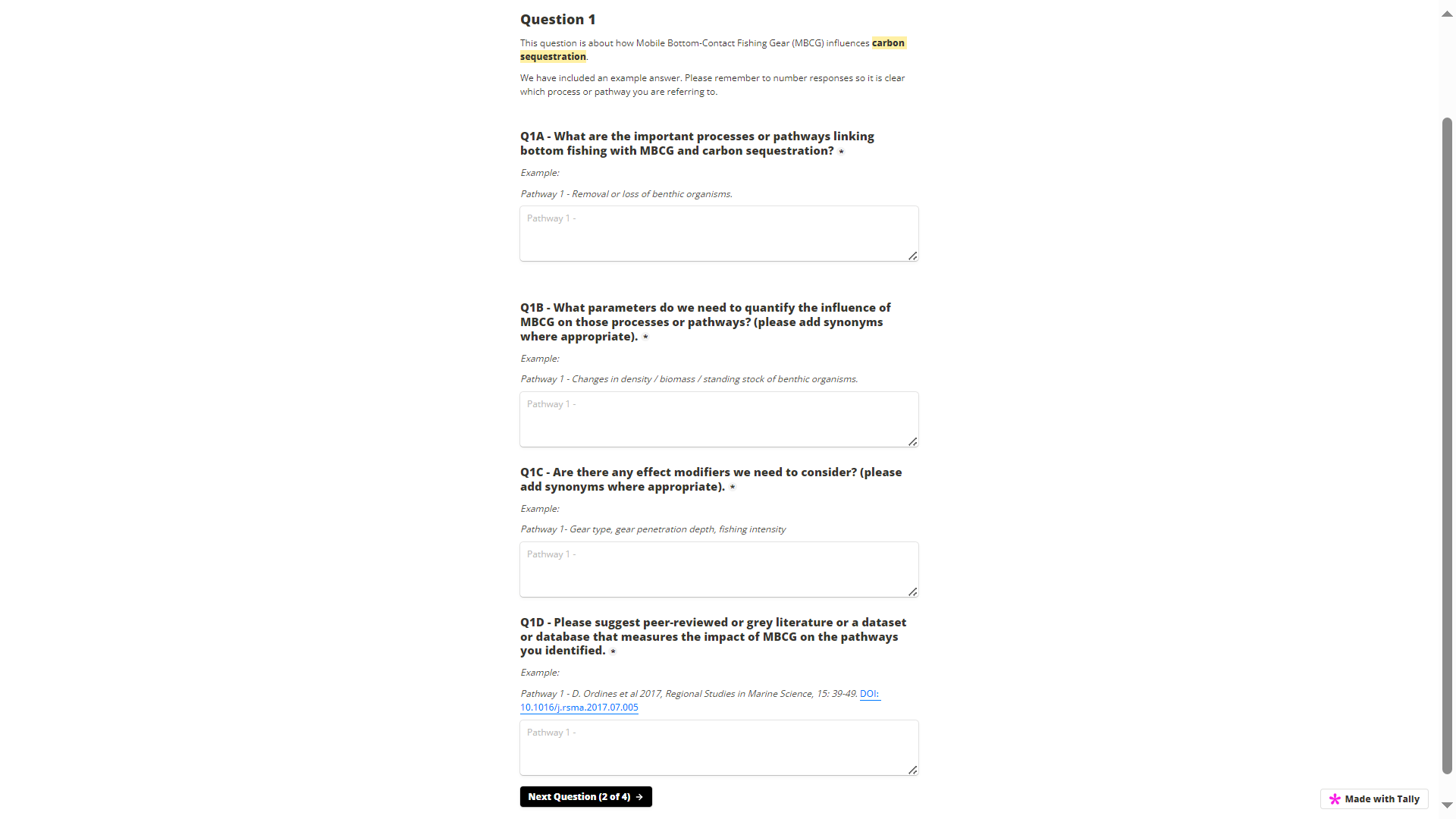


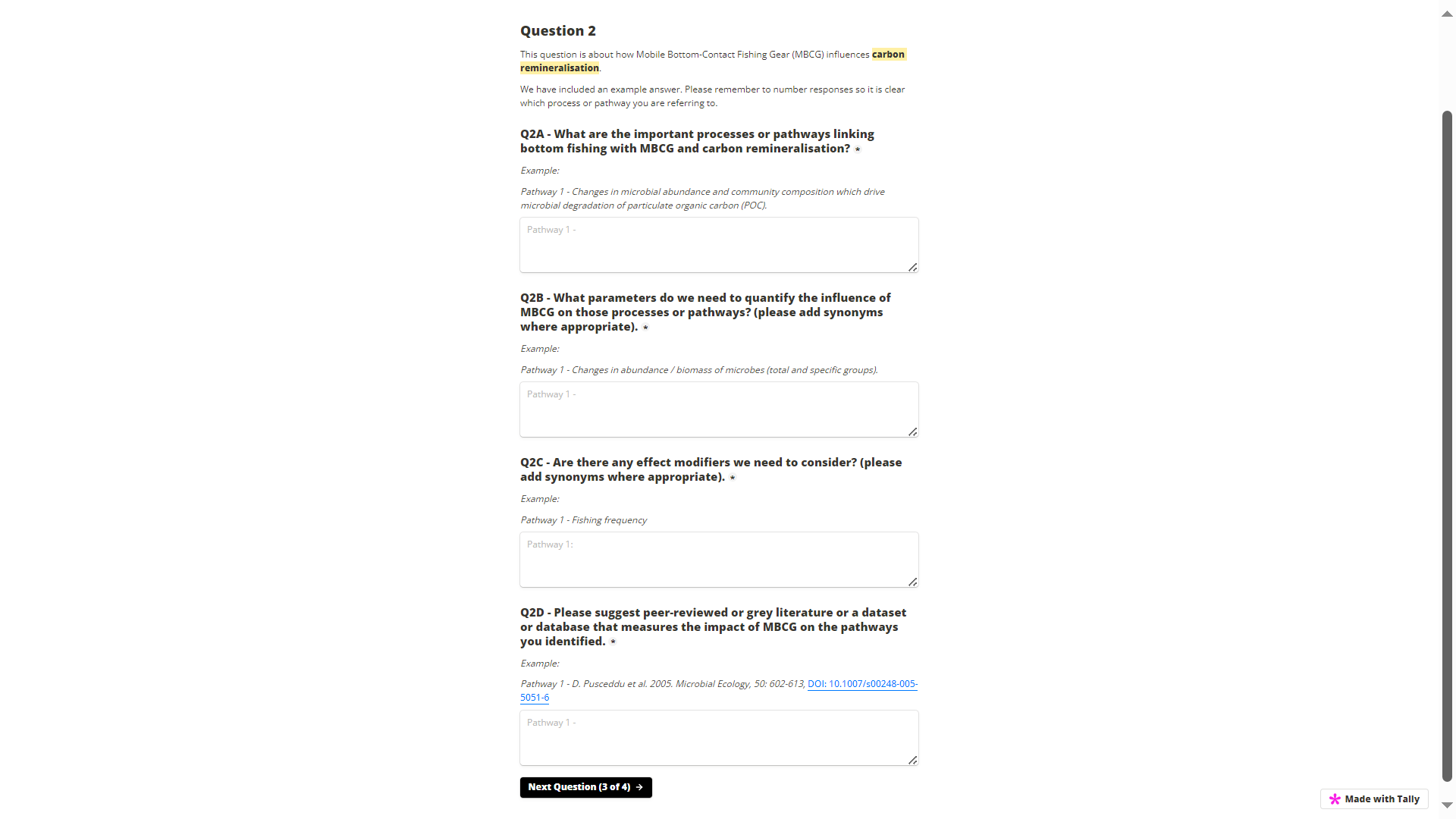


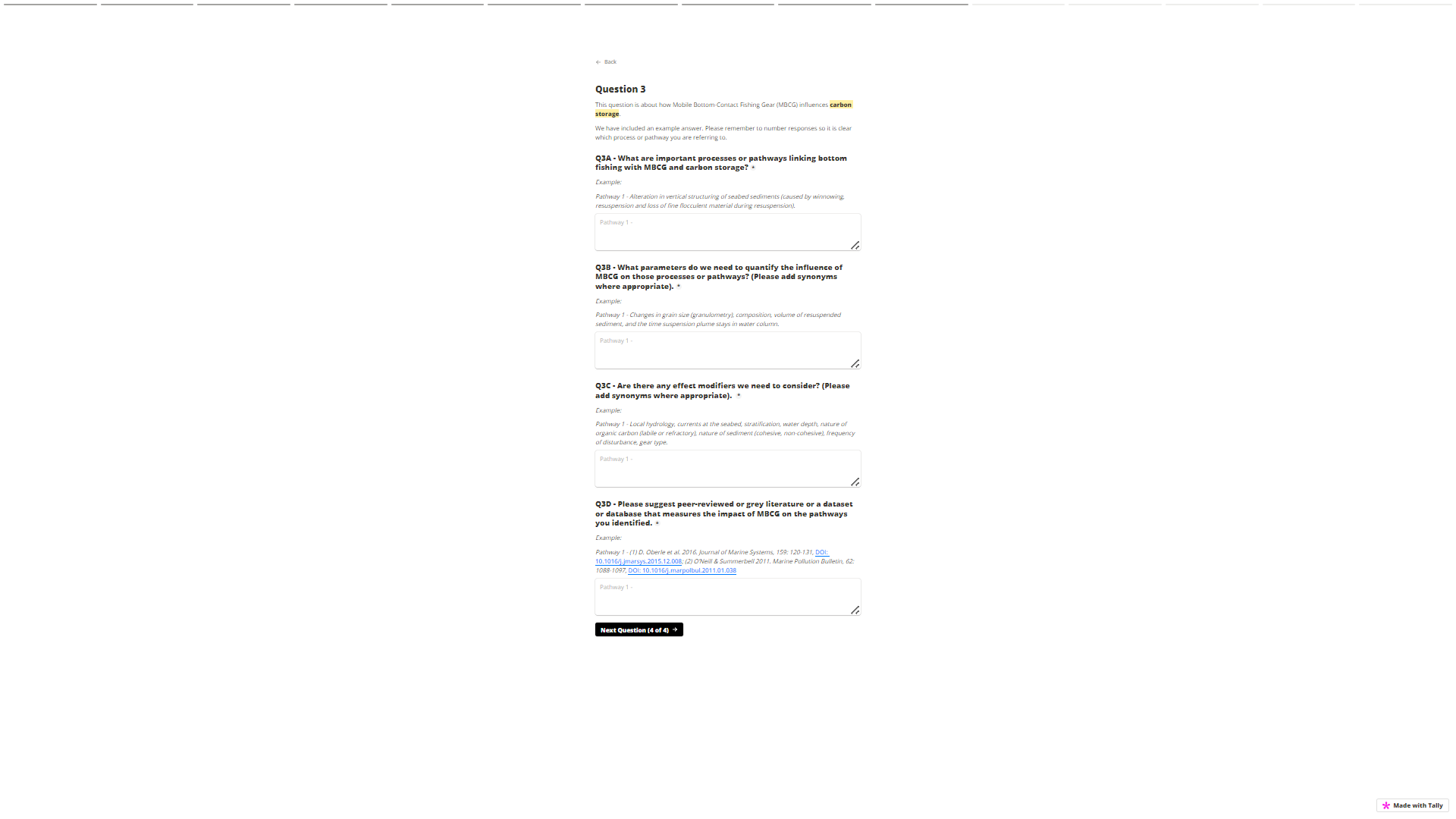


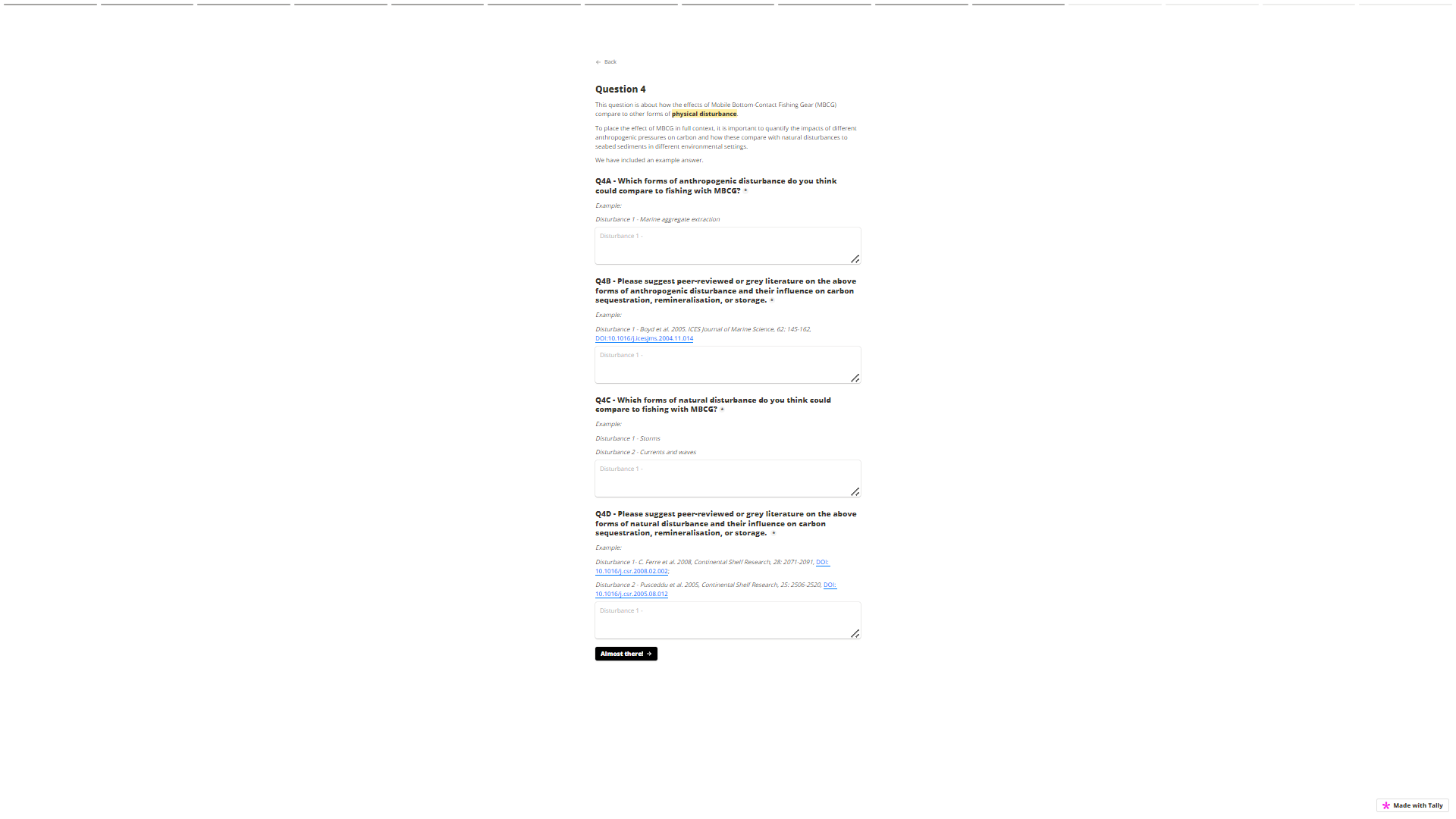


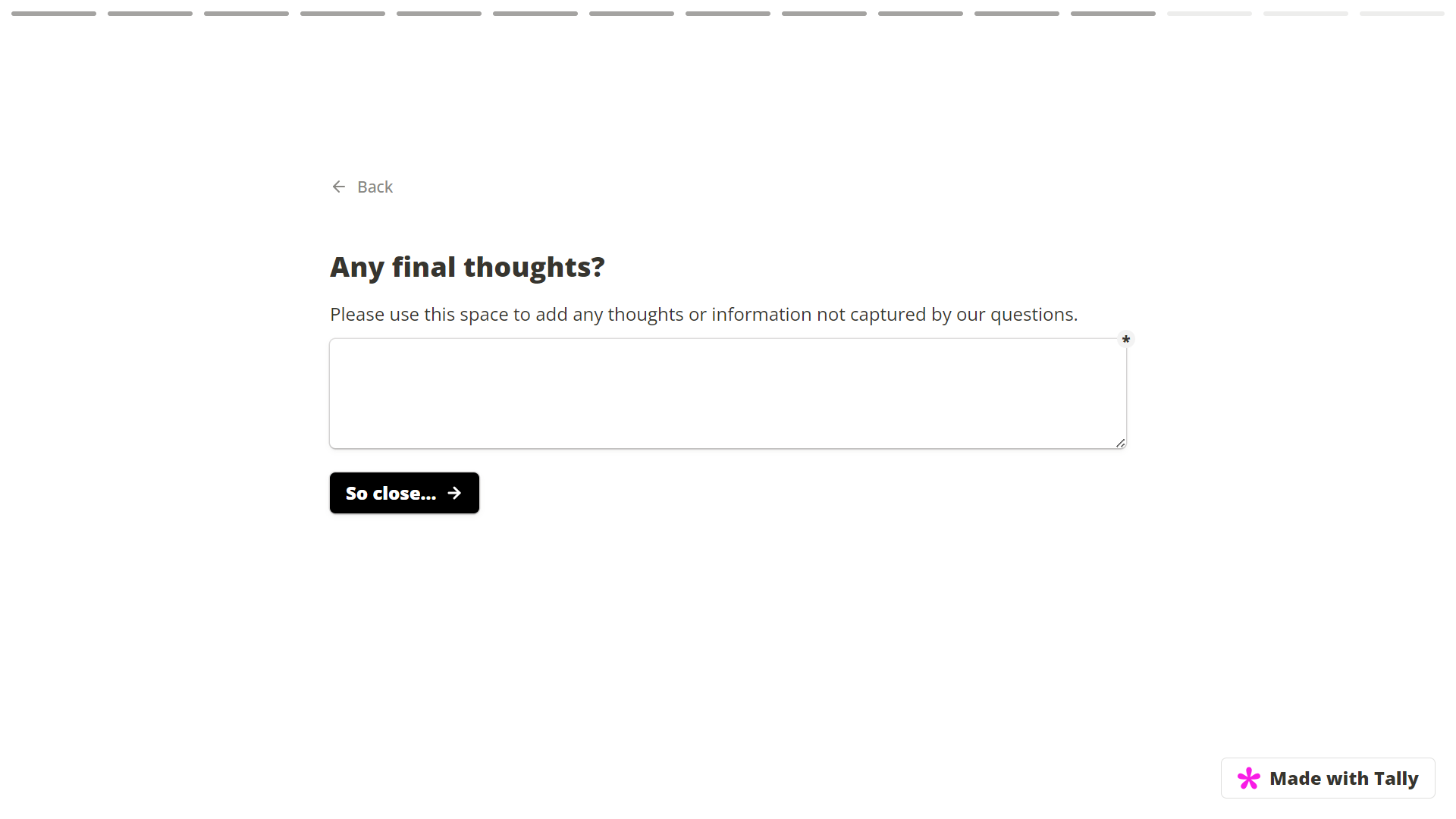


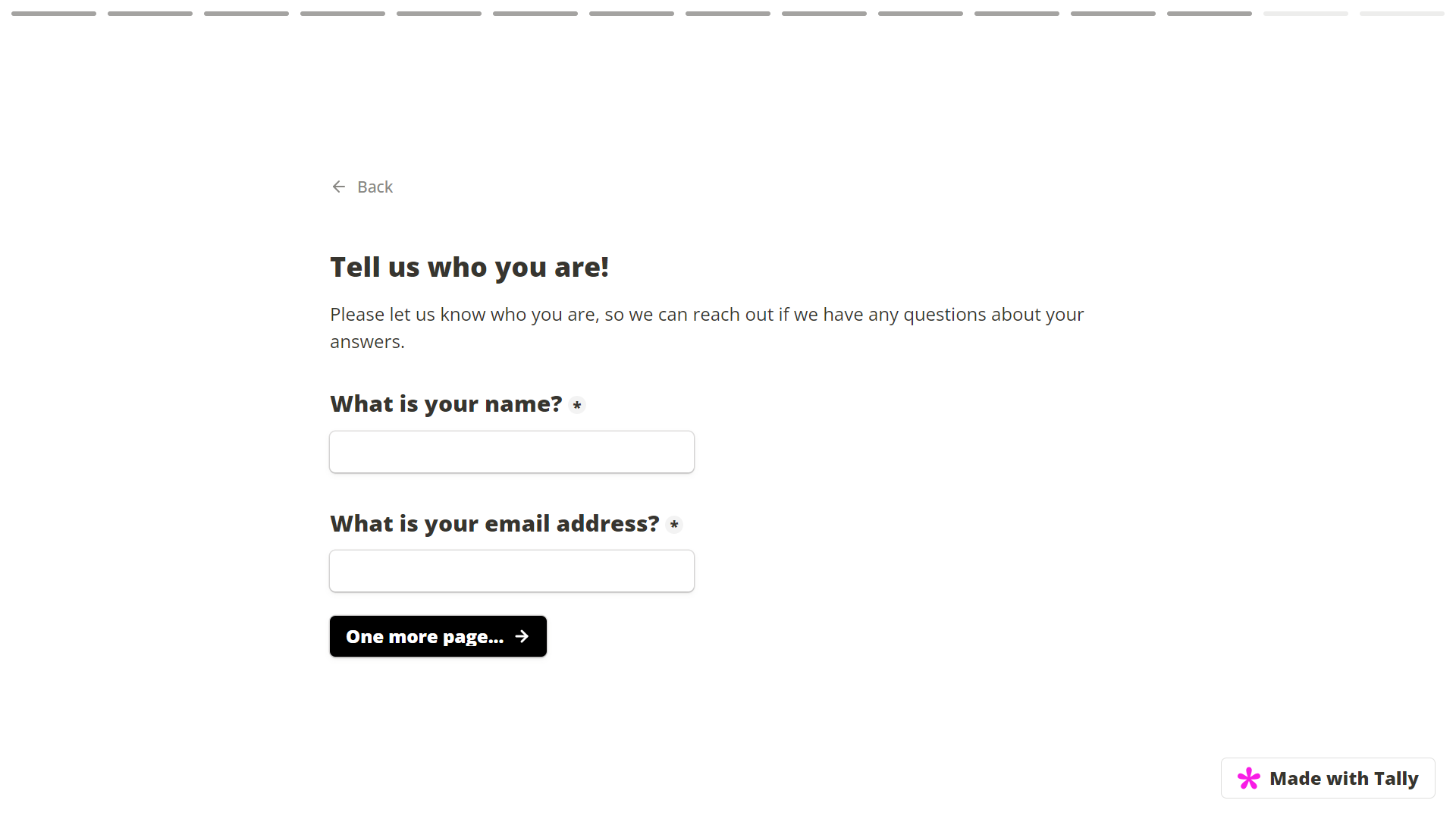


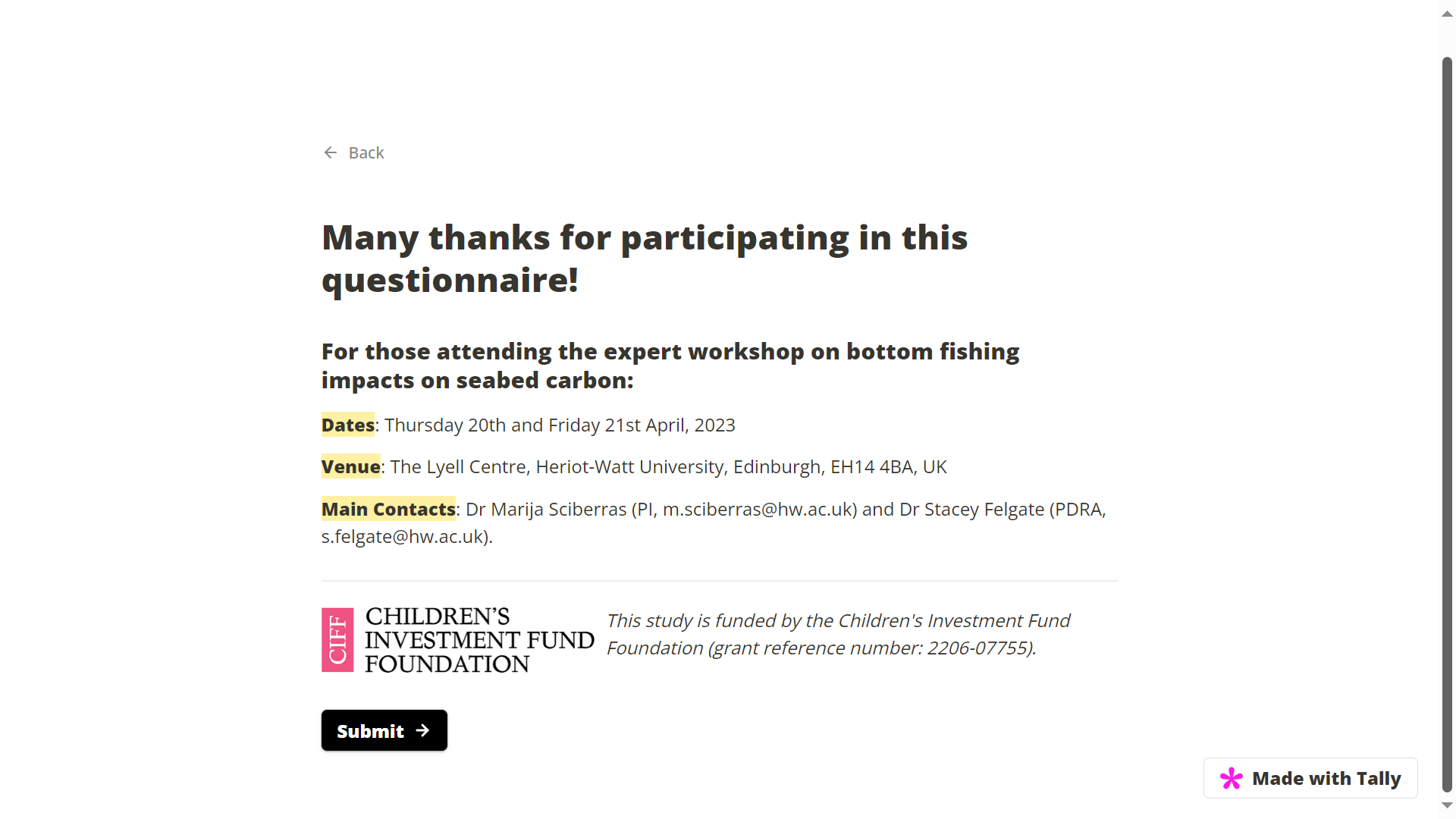

Supplement: Supplementary file 2 — Supplementary Material 2 [file 13750_2024_348_MOESM2_ESM.docx]
